# Notes and Comments

**Published:** 1922-09

**Authors:** 


THE
### HOSPITAL *and* HEALTH
REVIEW

### THE BRITISH MEDICAL ASSOCIATION CLIMBS DOWN.

IN our May issue we summarised the official report which consolidated the policy of the British Medical Association with regard to the voluntary hospitals and was prepared for presentation to the Representative Meeting at Glasgow. We criticised several of the proposals, and these were further discussed in our columns by correspondents, including the Chairman of the Hospitals Committee of the Association. We are pleased to notice that one proposal of the Report, to which we strongly objected, has been defeated. The Association demanded that a percentage of all payments made by or for patients (excepting private patients, who are not in question) should be passed into a fund at the disposal of the staff. We admitted the claim of the staff to a percentage of payments which exceeded the cost of maintenance, but held that contributions towards this cost should not be liable to the levy, as it would check subscriptions and endanger the whole voluntary system. As the report in another column shows, our view is endorsed by practically the whole of the medical staffs of the London teaching hospitals. We also disputed the Association's contention that patients making such contributions were the less fit subjects for charity or that the system altered the position of the honorary staffs. At the Glasgow meeting an amendment was carried, in spite of strong opposition, that a percentage should be levied only on payments made in part by rate aid or State aid and in other cases on payments in excess of the cost of hospital maintenance and accommodation. The untenable position, taken up in the first instance by the Association, is an example of that bias which seems inevitable in all combines, whether of professional or manual workers, when their interests are in question. The claim to levy a percentage on the small contributions towards maintenance was supported by the argument " In so far as patients pay they cease to be objects of charity." But the point is: " In so far as they cannot pay they are objects of charity." It now costs £3 or £4 per week to maintain a patient in hospital. When his weekly contribution, usually a matter of shillings, is deducted, he costs the hospital more than in the pre-war period, and, therefore, he has become more than ever an object for charity At the Association meeting, one of the representatives made the following important point: If a patient can pay £2 per week and the medical staff levy 8s. on that, this would really come from the other £2 contributed by the charitable public. We congratulate the hospitals, whose struggle for existence is so difficult just now, and also the Association, on the defeat of this dangerous proposal. If carried, it would have brought State control, with all its evils, distinctly nearer.

### ASYLUM REFORM.

THE Departmental Committee set up by the Ministry of Health to investigate the charges made against the administration of public mental hospitals by Dr. Lomax in his book "The Experiences of an Asylum Doctor," has presented its report. It finds that Dr. Lomax draws attention to some aspects of asylum administration which have long been recognised as admitting of improvement, but discredits most of his charges. His experience was derived from 19 months' service in one asylum (Prestwich) during the war, when conditions were abnormal. The suggestion that our mental hospitals are barrack-like buildings, and that patients are herded together without classification, is declared to be grossly exaggerated in the case of Prestwich, and untrue of mental hospitals generally. But the arrangements for the reception of new patients in Prestwich were inadequate. There is no ground for the allegation that systematic ill-treatment prevails in our asylums. The charge that patients are kept drugged and are purged with croton oil as a form of punishment is unproved. As to the allegation that patients are unnecessarily detained in institutions, there is no evidence that persons who have recovered their sanity are detained, but the Committee considers that if arrangements for after-care were improved a certain number of patients who did not really need institutional care could be discharged, provided that proper sheltered conditions were available outside. There is nothing to show that the facilities for surgical treatment are inadequate, or that every means is not used to restore the insane to health and sanity. There is room for improvement in the diet and in the means for employing and occupying patients. The Committee makes the following recommendations :—

(1) That for the future the size of mental hospitals should so far as possible be limited to accommodation for 1,000 patients.

(2) That in classification of patients account should be taken of the home conditions and surroundings from which they come.

(3) That the superintendent of a mental hospital should be a medical man, who should have undivided control of the medical and administrative work ; and that a small advisory board, preferably associated with the Board of Control, should be available for consultation by visiting committees when making these appointments.

(4) That the number of assistant medical officers should be increased, and that facilities for study-leave should be given.

(5) That the Departmental Committee on the Nursing Service should consider whether some distinction cannot be made between the two duties of mental nurses—namely, nursing proper and social duties—and that the hours devoted to the former should be relatively few, but that more time should be given to the latter, and that the present rigid system involving short shifts of duty should be discontinued.

(6) That the diet of mental hospitals requires improvement in the direction of greater variety.

(7) That the employment of patients could be improved by the appointment of a special officer at each institution as occupations officer.

(8) That the organisation of after-care work needs to be considerably strengthened and extended.

(9) That the possibilities of co-ordinating higher research work by concentrating it in a few fully equipped institutions should be considered.

(10) That Visiting Committees should be strengthened by the co-option of persons who have special qualifications and time to devote to the work, and that they should consider the authorisation of some small unofficial committee to visit the mental hospitals from time to time.

(11) That the Board of Control needs additional assistance to carry out its work.

---

### PREMATURE SANATORIUM DISCHARGES.

IT is impossible to be blind to the fact that sanatorium treatment is under a cloud. There are several reasons for this. In the first place, more was expected of the sanatorium than it could possibly achieve, and it is now suffering from the inevitable reaction of disillusionment. Another reason for the growing wave of scepticism as to the value of the sanatorium is the inefficiency with which many public sanatoriums are conducted. Time was when sanatorium treatment consisted solely of graduated rest and exercise in open air with a liberal dietary. But even this modest and out-of-date conception of sanatorium treatment is not always reflected in the conduct of public sanatoriums, and exercise is not graduated with sufficient care to meet the needs of the individual case. Thanks to the important advances made by Prof. Saugman and other Continental authorities, modern sanatorium treatment has become a highly elaborated system in which hydrotherapy, heliotherapy, the X-rays, artificial light, certain drugs, artificial pneumothorax and other forms of "lung-collapse treatment" play an important part. Under this new system the results achieved are often remarkable, the more so because many of the most dramatic and complete cures can now be effected in advanced cases. But the tragedy of the situation is this, that this highly skilled and technical system has barely secured even a precarious footing in this country, and the standard of treatment in many a public sanatorium is little different from that of a second-rate convalescent home. How unsatisfactory matters really are transpires in a paper published by Dr. J. B. McDougall in "Tubercle" for July, 1922. An analysis of 3,494 discharges from sanatoriums and tuberculosis hospitals to which the West Riding County Council sent patients between January 1, 1918, and October 29, 1921, showed that only 54·8 per cent. could be considered as regular and in order. Of the premature or irregular discharges 24 per cent. were for disobedience. No reason was given for the premature discharge in 26·9 per cent., and the chief factor in this class was that "they were, for one reason or another, not satisfied with modern methods of treating pulmonary tuberculosis." Considering that the methods practised in most sanatoriums are not modern, it is hardly remarkable that the patients in this class were dissatisfied. Domestic and economic factors accounted for 18 per cent. of the premature discharges, and many of the patients in this class would doubtless have been able to prolong their stay had an efficient After-Care Committee provided for the needs of their dependents. The conditions under which patients receive sanatorium treatment are often so far below the ideal that they are a travesty and a mockery. They will remain so until the whole subject is well ventilated without respect of persons.

### A SELF-SUPPORTING TUBERCULOSIS COLONY.

THE visit of the Minister of Health, Sir Alfred Mond, for the formal opening of the Cambridgeshire Tuberculosis Colony at Papworth, calls attention to an interesting scheme. The Colony includes 31 new cottages, two hostels, a nurses' home and various workshops. The President of the Colony, Sir Clifford Allbutt, explained that this is the first village settlement for consumptives, and that its function is not to rival or displace the sanatorium. Consumptives in all stages of the disease are admitted, and can have their families with them, so far as the number of cottages allows. The patients are employed in sign-writing, designing, boot-making, cabinet-making, printing, joinery, and trunk-making, and the workshops, he said, bring in a considerable revenue. Sir Alfred Mond described the settlement as unique; it provided not only treatment, but after-care and productive industry. In the workshops he had noticed the excellence of the work, and was glad to hear that there was a real market for it, and that the business was self-supporting without the need of charitable support either in the purchase or manufacture of the goods. It was hoped in time to establish more of these settlements. Statistics showed that consumption had declined in the past 80 years from 3,000 per million to 840 per million. Better housing was one of the essentials in combating the disease. He hoped that the example of Papworth would be followed in other parts of the country.

### THE DANGER OF GALL-STONES.

THE time has most fortunately passed when physicians prescribed opium and compresses for appendicitis, and took all the credit for the occasional recoveries which occurred in spite of this treatment. Unfortunately, the same cannot be said about gall-stones, and it is still the practice of many physicians to defer diagnosing gall-stones till an attack of acute biliary colic or definite jaundice has occurred. And even then many a patient is given only expectant treatment, which is but a euphemism and a paraphrase of Micawber's principle, that if you wait and do nothing something will turn up. Dr. Svend Hansen, pathologist to the "Kommunehospital" in Copenhagen, has lately published a serious indictment of the temporising physician. During a period of 21 months, he made an exhaustive search for gall-stones among all the adults examined post mortem, and among 1,191 he found 293 with gall-stones. In other words, 25 per cent. harboured gall-stones! In 43 per cent. these gall-stones had given rise to no inflammatory changes in their surroundings. They were, in fact, harmless guests. But in the remaining 57 per cent. they had caused morbid changes, and in 14 per cent. they were the cause of death. In the same period only 26 post-mortem examinations were made on persons dying of appendicitis. The facts that 40 patients died of their gall-stones in this period, and that nine of them died in medical wards, are not satisfactory. Dr. Hansen's indictment is intended primarily for the physician who is satisfied with the vague diagnosis of dyspepsia, and suffers from a rooted dislike to passing a patient on to a surgeon. But here and there the surgeon himself comes in for criticism, and the surgeon who performs a laparotomy and asserts that no gall-stones are present after he has palpated the gall-bladder, is reminded that this verdict is frequently reversed a few hours later in the post-mortem room. Gall-stones are slippery, elusive things, and even the pathologist may overlook them unless he slits open every biliary passage. At the present time, when the stage is teaching the public to regard the surgeon as a vampire who wages ruthless warfare on healthy appendices in order to extort big fees, it is well to bear in mind that gall-stones, at any rate, take daily toll of lives that might have been saved had the timely aid of a surgeon been invoked.

### THE MATERNITY BONUS IN AUSTRALIA.

THE "Medical Journal of Australia," of June 3, publishes an interesting article with regard to the operation of the "baby bonus" of £5 given in Australia. It was hoped that this maternity bonus, given to a woman at this critical period, by relieving her of financial difficulties would improve her health, increase her comfort and also benefit the baby. In time the law was extended to babies born dead.

The "Medical Journal" states that, whatever the intention of the framers of the Bill, "it has failed completely and unquestionably in reducing the dangers of childbirth to mother and infant." It is considered that "the greater part of three-quarters of a million pounds sterling is misused to the detriment of the welfare of the community every year. This waste is a very serious matter from the point of view of preventive medicine." The Federal Government has been urged by members of the medical profession to utilise part of this ill-directed bonus to provide the better equipment of obstetrical hospitals, and especially the enlargement of the Royal Hospital for Women in Sydney, and its adequate adaptation as a teaching institution. In order that these and other measures might be encouraged by financial assistance from the £750,000 now "squandered," it would be necessary to adjust the relations between the Federal Government and the State Governments. In such a way it is considered that Australian mothers and their new-born infants would be really assisted.

### REFUSE DISPOSAL.

THE problem of the disposal of the refuse of our large industrial centres is becoming increasingly acute. In the Administrative County of London, for example, the aggregate amount of refuse to be disposed of annually is 1,500,000 tons, or about 5,000 tons for every working day. This has to be collected from an area of 117 square miles, with a population of approximately 4½ millions. Of this huge quantity collected by 1,320 vehicles, 80 per cent. is merely dumped on low-lying land and in disused pits, at a cost of about £600,000 per annum.

Many of these dumps are a serious nuisance and a grave danger to public health. The refuse, especially in warm weather, provides excellent breeding ground for rats, flies and other germ-carriers. The system also means the waste of a number of valuable by-products. At the present time practically all the available dumping-grounds are in the hands of a few contractors. The London problem is similar to that now engaging the attention of a number of local authorities. For that reason some of the conclusions arrived at during a conference recently held under the chairmanship of Mr. I. G. Gibbon, Assistant Secretary of the Ministry of Health, are of interest.

Mr. Gibbon considered that the time was not opportune to discuss any comprehensive scheme of disposal involving heavy capital expenditure. The separation of refuse, its pulverisation and the refuse destructor all necessitated such expenditure. At the same time, if railway rates were lowered it was probable that farmers would be willing to buy pulverised refuse, which has considerable value as a manure.

Much, however, could be done by periodical inspection of the dumps to render the tips relatively harmless. At one place in the neighbourhood of London the tip is a breeding-place, not of the usual plague of rats, but of rabbits. The example of St. Marylebone, where a salvage scheme for dealing with refuse has been adopted, to the great advantage of the borough, might also be followed. By such economies, conditions of refuse disposal might be greatly improved.

### A STATESMAN'S SPEECH.

ONE of the most statesmanlike speeches ever delivered in connection with public health was that made by Major-General J. E. B. Seely as President of the Congress of the Royal Sanitary Institute at Bournemouth. He insisted that he had no sympathy with those who wish to cut down expenditure on the public health services. "We simply cannot afford to save money on these subjects." He objected to the proposed restriction of inspection of the 6,000,000 children in the elementary schools, for, he urged, the school medical service was not only saving thousands of children's lives every year, but was laying the foundation for a healthy nation in years to come. He lamented that in certain districts there was an inclination to go back, and mentioned that out of 317 education authorities 76 had made no provision for the treatment of dental defects, 26 had entirely neglected the treatment of defective vision, and 95 had made no provision for treatment of diseases of the throat and nose, particularly common in children. His view that the time lost because of sickness or accident was of serious impost to industry, was supported by his statement that we were losing in the insured population alone the equivalent in output of 278,000 persons all through the year.

His commonsense views on the milk question might well be studied by some faddists, for he suggested that there were three ways by which we could obtain more milk and cleaner milk: (1) The consumer should demand clean milk and protect it from uncleanliness in his own house—a source of contamination too often overlooked; (2) it is to the interest of the dairy trade to conduct its business in a clean and scientific manner; (3) the sanitary authorities should put into force their powers of control regarding adulteration, uncleanliness and infection.

### THE SALE OF UNSOUND FOOD.

AT a conference of representatives of Metropolitan Borough Councils held at the Islington Town Hall on July 18, to consider the question of the sale of unsound fruit, vegetables, tinned foodstuffs, &c., by wholesalers to costermongers, and the subsequent sale to the public, and the steps to be taken with a view to stopping this practice, Dr. G. Clark Trotter (M.O.H., Islington) said that the difficulty experienced in Islington in the tracing of unsound food and of taking proceedings against tradesmen who dealt in the cheap packages disposed of by the wholesaler could be avoided if the wholesalers were required to sort the food before offering it for sale. The difficulty was aggravated by the wholesaler in some instances putting up a notice in his sale-rooms that the goods offered were sold on condition that they were to be sorted by the purchaser. The extent to which this trade had been carried on in London showed the need for some amendment of the law to enable unsound food to be dealt with in bulk before its distribution amongst costermongers and small retailers. A resolution was carried unanimously: "That the attention of the Minister of Health be drawn to the necessity for the law relating to the sale of unsound food being amended by the addition of the following words at the end of section 47 (3) of the Public Health (London) Act, 1891, 'and had taken reasonable steps to ascertain that the article was not in such a condition as to be liable to be seized and condemned under this section.'"

### THE SMOKE ABATEMENT BILL.

THIS Bill, introduced into the House of Lords, has received a second reading, and now stands over for consideration in the autumn. Lord Newton, whose labours as Chairman of the Committee on the smoke abatement question were acknowledged by Lord Onslow, the Minister in charge of the Bill, welcomed the measure as an expression of the Government's good intentions, but added that he felt rather like a man who had been promised a passage to America, and has in fact been given enough money to take him to Liverpool.

The Bill includes in the smoke nuisance not only black smoke, but also gritty particles, soot, and ash ; it throws the onus of proof that he has used the best means of preventing a nuisance on the person charged, provides for heavier fines, and authorises the county councils to step in and act if the smaller authorities default. It does not give effect to the more heroic recommendations of Lord Newton's Committee, that the powers of the small local authorities should be transferred to the county and county borough councils, and that the Ministry of Health should have power to insist upon the provisions of the Bill being carried out. Lord Newton will have the opportunity himself of raising these more controversial questions on the Committee stage of the Bill ; he has also been invited by the Government to offer practical suggestions as to how the smoke "standards," which his Committee recommended, can be determined and enforced.

### THE MILK AND DAIRIES ACT.

A MERE postponement of the operation of the Milk and Dairies Act of 1915 would have been a poor achievement. But that is the main purpose of the Bill which has just become law—the avoidance of any fresh public expenditure being the dominant consideration.

The Milk and Dairies Amendment Act, accordingly, in addition to the postponement provisions, gives power to the local authorities to revoke the registration of any retailer who is guilty of any act or default dangerous to the public health, with safeguards against arbitrary action by the requirements that notice is to be given to the retailer and the authority of a Court of Summary Jurisdiction obtained, and with a right of appeal to Quarter Sessions.

Another clause imposes a heavy penalty on any person who knowingly sells the milk of a cow suffering from tuberculosis of the udder, which would, presumably, apply only where the ordinary farmer would be aware of the disease in the absence of an expert diagnosis. The provisions of two Orders of the Food Controller which, as such, lapse on September 1, are continued. One protects the designations "Certified Grade A" and "Pasteurised," but it is understood that the Order for which the Act provides will so modify the conditions for Grade A milk as to bring its protection within the compass of every careful farmer and dairyman. The other provision makes it easier to obtain a conviction for adulteration by making it an offence to add water to milk intended for sale.

### NATIONAL HEALTH INSURANCE MEDICAL BENEFIT.

THE National Health Insurance Act, 1922, adds to the Statute Book yet another amending Act affecting the finances of Health Insurance. Its principal provision is that which transfers, for a limited period, to the Benefit Funds of Approved Societies, that part of the cost of Medical Benefit which was being borne by the Exchequer in excess of the proportion of two-ninths originally contemplated as the Exchequer contribution by the Act of 1911. The Benefit Funds are re-credited with one-third of this excess from the Central Fund, which is a fund available in certain conditions for meeting deficiencies of Approved Societies. The question of the effect of this new provision on Medical Benefit finance and on the relations between the Approved Societies and the medical profession was dealt with in a specially contributed article in our last issue.

### THE EDINBURGH SCHOOL OF SOCIAL STUDY AND TRAINING.

IT is now recognised that theoretical and practical training are required by all who desire to qualify themselves for social work, whether as officials or as voluntary workers. In response to this need, an Association has been formed consisting of representatives of the University of Edinburgh, and of all bodies in the district interested in promoting social study and training. The objects are to provide, under the auspices of the University, courses of study and training for persons desirous of (*a*) preparing themselves for the duties of members of local governing bodies ; (*b*) taking part in social work, whether as volunteers, or as official secretaries and organisers of such work ; (*c*) engaging as officials in national and municipal administration (*e.g.*, Poor Law, Insurance, Public Health, Housing, Employment Exchange, Educational Work, After-Care Work, Day Care of Children, &c.) ; (*d*) qualifying themselves as Welfare Workers in Factories, or as Health Visitors, and for similar employments ; (*e*) engaging in the social welfare work of churches. The regulations of the course of study to qualify for Health Visitor will be found in our Educational Supplement (p. 374).

### HEALTH EDUCATION IN THE UNITED STATES.

THE American health authorities, whatever their shortcomings in practical public health administration and in scientific registration, are certainly far in advance of Great Britain in harnessing publicity to public health. Last November a campaign against cancer was opened with a lecture "broadcasted" by radiophone from Denver. Early this year the United States Public Health Service and the New York Health Department began to send out "five-minute health talks." The United States Public Health Service issues regularly snappy paragraphs called "Health Nuggets" for weekly journals and other periodicals. Some of these are valuable. Others bear traces of commercialism, as, for example, the following: "Uncle Sam may owe arsphenamine (salvarsan) to Germany, but he has improved it a lot since he took over its manufacture some years ago. To-day it and its fellows pass tests that are twice as rigid, which means that the drugs themselves are twice as safe as they were before." The heading of this puff is "Who's Efficient Now ?" Some critics are inclined to abuse the British Ministry of Health for being old-fashioned, but most of us have reason to be grateful that, so far, American methods have not been imitated in Whitehall.

### UNIONS AND DIVISIONS IN ASYLUM STAFFS.

A CURIOUS position has arisen at Cardiff in connection with the conditions of work of the staff of the Whitchurch Mental Hospital. Recently the Conciliation Board made an award between the Asylum Workers' Union and the Mental Hospitals Association. The Union represents about 80 per cent. of mental hospital workers. Dr. Goodall, the medical superintendent, reported that the award increased the hours of the staff from 54 to 60 a week, reduced annual leave from four weeks to three, and slightly altered the pay of *new* staff members. When Dr. Goodall began to enforce the award, a deputation of the staff arrived, saying that the award was a bombshell to them, because none of them was a member of the union. Their body is the Municipal Employees' Association. The organiser of this association later explained to Dr. Goodall that the association favours a 48 hour week, could not accept a 60 hour week, and was unable to work it. He has therefore postponed action till September, when more information would be available. The Asylum Workers' Union, it is interesting to note, has accepted the award, and is now balloting its members upon it.

These facts are of general interest, because once again they indicate the complications in mental hospital management arising from the different bodies to which the rank and file of the staffs belong. Before the war there were at least two, the one established and moderate, the other new and extreme. It is, probably, because of their competing claims upon the support of the asylums' staffs, and of the unpleasant incidents arising out of the activities of one of them, that asylum workers, like these at Whitchurch, have joined the Municipal Employees' Association. But while the staffs divide their membership between two or three different bodies pursuing divergent and even contradictory programmes, general concord and agreement becomes well-nigh impossible. The authorities will not know with whom to deal, settlements of points in discussion will remain sectional, and the goodwill and co-operation of staffs and their employers will be hindered everywhere. An intolerable position must be avoided in the interests of the staffs themselves; and a conference seems to offer the only solution of the difficulty. For this reason Dr. Goodall's delay is welcome and judicious. Unions which divide their members are a source of weakness not to be endured.

### CONVALESCENTS IN GENERAL HOSPITALS.

THE Board of Management of the Royal Victoria Hospital, Newcastle-upon-Tyne, has reluctantly decided to refuse to admit cases of surgical tuberculosis, unless provided with a guarantee that there is a suitable place to which they can go after operation. The reason, of course, is that such patients occupy beds for a much longer period than other surgical cases, and yet need after operation little more than food and rest. It is asserted that 10, or even 12, patients of other surgical classes could be treated in the time occupied by one case of surgical tuberculosis, which cannot be sent home after operation. Now, the Royal Victoria Hospital has a long waiting list, and is therefore specially alive to the need for discharging patients as soon as possible. For the cases no longer to be admitted without a guarantee a convalescent home is the proper place, but the hospital is unable to provide the cost of one at present. It remains to be proved whether after-care can best be secured by an institution attached to the infirmary, or whether such a convalescent home should be supported by a separate body and a separate fund. A case could be made for a special hospital for them, which would imply a surgical centre in Newcastle, and one or more convalescent homes in the country. Special hospitals exist for children suffering from this disease, but if the cases can be dealt with at the Royal Victoria Infirmary, a convalescent home devoted to them would suffice. It is impossible to impugn the grounds of the Infirmary's recent decision, and perhaps its drastic action may arouse sufficient public interest to raise the necessary sum for the new home. For a general hospital ward is obviously not designed for cases requiring long periods of convalescence.

### THE HOSPITAL SAVING ASSOCIATION.

A HOSPITAL Saving Association has been formed and registered by Messrs. Morris, Veasey & Co. By licence from the Board of Trade the word " Limited " has been omitted from the title. The subscribers to the memorandum of association are Lord Hambleden, Lord Goschen, Lord Knutsford, Sir Alan Anderson, Hon. Sir Arthur Stanley, Sir Edward Penton, and Mr. Stuart de la Rue. The objects of the Association, which is not formed for profit, is limited by guarantee, and without a capital divided into shares, are to organise a system of continuous subscriptions from eligible members for the support of hospitals; to relieve contributors or their dependents when under treatment from hospital charges; to repay to hospitals the cost of the maintenance in hospital of contributors and their dependents; to make grants or loans to hospitals; and to maintain facilities for hospital treatment for contributors and dependents. The first members of the executive council are to be nominated by the subscribers to the memorandum of association. No call on members shall exceed 5s., and all calls in any one year shall not exceed £1 per member. The number of members is unlimited, and each member agrees to contribute up to two guineas if the association is wound up.

### SHOULD PATIENTS SUPPLY THEIR OWN BREAD?

IN reply to vague rumours that the diet of patients in the Keighley Victoria Hospital is insufficient, and that the patients have " to find most of their own food," the secretary has issued both a denial and a possible explanation. It appears that absence of variety has led to patients being allowed to have " bread brought from home, as well as their own fresh butter. Fresh eggs are also allowed, and if patients wish for extra cups of tea after dinner, or instead of cocoa for supper, they have been allowed to provide their own tea and sugar for it." These privileges are thought to have led to the rumour, and their existence calls, we think, for a word of comment. While the rules requiring hospital patients to provide this or that necessary vary from one hospital to another, it is not usual nowadays for the patients to be required to supply the simplest articles of diet. That they should themselves wish to provide fresh bread argues that in this respect the hospital's diet is defective. Butter may still be regarded as a luxury in some institutions, though we were glad to see that a London hospital lately reverted to its provision, as was its custom before the war. Tea instead of cocoa for supper is not an unreasonable alternative, and though linen, tea, sugar and butter are sometimes required of patients, the best rule is that obtaining at St. Thomas's, where patients are not required to provide any article of food. Fresh bread, in any event, cannot be regarded as a privilege. We are therefore of opinion that the committee of the Victoria Hospital, Keighley, should instruct its secretary to compare the present diet with that of other hospitals. If this be done, we fancy that some of the privileges sought at present by the patients of Keighley will no longer be in request, and that an avoidable prejudice against the hospital will be removed.

### PAYMENT OF HOSPITAL MEDICAL STAFFS.

THE following resolutions have been passed at a conference of representatives of the medical staffs of the following London teaching hospitals :—St. Bartholomew's, St. George's, Guy's, King's College, St. Mary's, Middlesex, St. Thomas's, University College, Westminster, and the Royal Free :—

1. That under present conditions, in the case of patients who contribute towards, but pay not more than, the whole cost of their maintenance* in a voluntary hospital, it is undesirable that any portion of such contribution should be allocated to a staff fund. (Adopted with no dissentient.)

2. That when the State, a municipal or other public body, pays towards the accommodation, maintenance, and treatment of a patient, or group of patients, in a voluntary hospital, a percentage of such moneys should be allocated to a staff fund. (Adopted, Guy's, Middlesex, and University College dissenting.)

3. That when a special clinic is held by a member of a hospital staff for the treatment of patients sent by the State, a municipal or other public body, the member of the staff taking such a clinic should be adequately paid. (Adopted, Guy's dissenting.)

### THE TROUBLE WITH OPTICIANS.

BY the time that we go to press it will probably be clear whether the hospitals are likely to be affected by the dispute in the optical industry. At the time of writing the Conciliation Department of the Ministry of Labour is trying to bring the parties together. A stoppage would have unfortunate results, for hospital supplies of spectacles and glasses would be interrupted. The West End firms of opticians are chiefly involved, and allied unions may be brought in. Not only ophthalmic hospitals are concerned, for there is probably no out-patient department which does not employ spectacles, except in those few special hospitals that treat quite different diseases. Industry now affects hospitals at so many points that their financial troubles are always likely to be complicated by trade disputes with which, at first sight, they appear to have nothing to do.

### EXTENSIONS AT UNIVERSITY COLLEGE HOSPITAL.

WORK has now begun on the vast extension scheme for University College Hospital, which the munificent gift of £440,000 from the Rockefeller Foundation has made possible. The first part of the building programme includes a new Nurses' Home ; a six-storied obstetric block, with extensive equipment ; an enlargement of the medical school, including operating theatres ; and new quarters for the resident medical officers. Houses in University Street and Huntley Street are to be pulled down, and under the latter a tunnel will connect the new buildings to the old. The first part of the building scheme should be completed in 1924. The remaining extentions will include an open-air ward for septic cases and further research laboratories. On the first part of the scheme £250,000 is being spent. The architect of the extensions is Mr. Paul Waterhouse, P.R.I.B.A., acting with Mr. George Hornblower, consulting architect to the hospital. The reconstruction scheme will involve the conversion into wards of the present nurses' home, and will provide thereby 150 additional beds for patients. The maintenance of the new buildings is expected to cost an extra £20,000 a year. It will be recalled that the idea of the Rockefeller Foundation in making the grant was to establish a "Model Medical School" in London, in which new ideas and new methods of treatment could be devised and tested at a centre of propagation for the Empire.

### QUEEN MARY'S MATERNITY HOME.

LAST month the inmates of the Queen Mary's Maternity Home, temporarily lodged at Cedar Lawn, Hampstead, were transferred to the new Home built under special plans out of the surplus money placed at the disposal of Her Majesty by the governing bodies of Queen Mary's Needlework Guild and other societies. It has been erected under the advice and superintendence of the Ministry of Health, in order that it may serve as a model maternity home. The "Architects' Journal" states that the result is "a quiet brick building of Queen Anne character, designed to bear as homely an atmosphere as is consistent with the most modern ideas of hospital construction and sanitation." Fifteen patients can be accommodated, in wards containing beds for four, three and one, and all these rooms face south. There are also nurseries for eight

* By the maintenance is understood the cost per bed as calculated for the purpose of the return made by each hospital to the King's Fund.

older infants whose mothers are in the Home. The latest views with regard to maternity homes will be found in an article published in the July issue of this review, contributed by one of those most responsible for Queen Mary's Home.

### A DISCERNING FOUNDER.

A GENERATION ago the needs of the poorer middle classes were a less familiar topic than they are to-day; but it was as far back as 1895 that Sir Alfred Yarrow, the engineer, built and endowed the Yarrow Convalescent Home at Broadstairs, expressly to provide change of air for the children of professional people. Fifty boys and fifty girls are accommodated at a charge to their parents of 10s. a week, though the average cost per week last year was £2 6s. 6½d. Sir Alfred, who is now in his eighty-first year, and was present at the last Founder's Day Sports, has lately added largely to the endowment. The committee of the Home is now associated with the Institution of Civil Engineers. The staff consists of a matron and trained nurses.

### THE SPIRIT OF THE STAFF.

THE letters of gratitude received from patients, from the nature of things, rarely strike an original note, though they are excellent testimony to the spirit in which a hospital is conducted. Lately, however, a patient at the Devonshire Hospital, Buxton, wrote an excellent letter to Mr. T. B. Harrison, the hospital secretary. In this the writer, Mr. Joseph Connor, of Manchester, advised anyone "who had lost faith in humanity to go to the Devonshire Hospital, Buxton, where there is a great spirit of goodwill and chivalry, the like of which I have never seen before." This is well enough, but, the letter continued: "The more unfortunate are always helped by those on the road to recovery, and the unfortunates will, in their turn, help others who come later." This is remarkable, because, while a hospital staff is often praised for its kindness and attention, to single out fellow-patients for similar praise is much more rare. Yet surely in the ideal hospital patients and staff create the atmosphere of sympathy and understanding between them.

